# Adversidades no período intrauterino e neonatal interferem na inteligência?

**DOI:** 10.11606/s1518-8787.2025059006206

**Published:** 2025-06-16

**Authors:** Janielle Ferreira de Brito Lima, Rosângela Fernandes Lucena Batista, Liliana Yanet Gómez Aristizábal, Cecilia Claudia Costa Ribeiro de Almeida, Vanda Maria Ferreira Simões, Antônio Augusto Moura da Silva

**Affiliations:** I Universidade Federal do Maranhão. Departamento de Enfermagem. São Luís, MA, Brasil.; II Universidade Federal do Maranhão. Departamento de Saúde Pública. São Luís, MA, Brasil.

**Keywords:** Retardo no Crescimento Fetal, Fatores Socioeconômicos, Inteligência

## Abstract

**OBJETIVO:**

Investigar os efeitos do retardo no crescimento intrauterino e da situação socioeconômica de nascimento no desenvolvimento intelectual.

**MÉTODOS:**

Estudo realizado com 313 participantes de uma coorte de nascimentos de São Luís, Maranhão, avaliados ao nascerem e entre 18 a 19 anos. Utilizando modelagem de equações estruturais, as variáveis do nascimento e primeiros anos de vida (idade materna, escolaridade materna, escolaridade paterna, ocupação do chefe da família, renda familiar, ganho de peso materno gestacional, retardo no crescimento intrauterino, baixo peso ao nascer e tempo de aleitamento materno) e a escolaridade aos 18 e 19 anos foram testadas como determinantes do quociente de inteligência aos 18 e 19 anos.

**RESULTADOS:**

O retardo no crescimento intrauterino não apresentou efeito total (p-valor = 0,957), direto (p-valor = 0,184) ou indireto (p-valor=0,108) sobre o quociente de inteligência aos 18 e 19 anos. A situação socioeconômica no nascimento apresentou efeito total positivo de 0,406 desvio-padrão (p-valor < 0,001) sobre sua média, correspondendo a elevação de 4,54 pontos no quociente a cada elevação do nível socioeconômico no nascimento. Esse efeito não foi mediado pelas demais variáveis explanatórias inclusas no modelo, contudo, identificou-se efeito direto positivo de 0,416 (p-valor < 0,001) da situação socioeconômica sobre a escolaridade, que apresentou correlação positiva (CP = 0,439; p-valor < 0,001) com a inteligência. A idade materna no nascimento também apresentou efeito positivo direto de 0,116 desvio-padrão (p-valor = 0,042) sobre a média do quociente de inteligência aos 18 e 19 anos, correspondendo ao incremento de 1,30 pontos no quociente a cada elevação de 1 ano na idade materna no nascimento.

**CONCLUSÃO:**

Maiores níveis socioeconômicos no nascimento geraram efeito positivo direto de longo prazo sobre a inteligência e escolaridade do participante, elevando a média do QI e o nível de escolaridade aos 18 e 19 anos. Identificou-se também correlação positiva entre as duas variáveis.

## INTRODUÇÃO

O desenvolvimento intelectual tem sido amplamente estudado devido ao seu impacto em resultados socialmente relevantes, como sucesso profissional e pessoal^1^ ^)-(^ ^3^. O desenvolvimento da inteligência parece envolver aspectos genéticos^4^, neurofisiológicos^5^, ambientais^6^ ^),(^ ^7^ e relacionados à experiencias adversas vivenciadas da concepção à idade adulta^8^ ^)-(^ ^10^.

Adversidades vivenciadas no período intrauterino e neonatal têm sido associadas ao desenvolvimento intelectual ao longo da vida. Variáveis como situação socioeconômica (SES) da família^6^, aleitamento materno^11^, baixo peso ao nascer^12^ e retardo no crescimento intrauterino (RCIU) ^(^ ^12^ ^),(^ ^13^ parecem interferir no desenvolvimento neurológico e níveis de inteligência.

Dentre essas adversidades, o RCIU se destaca pelos riscos que oferece a formação e maturação cerebral^13^. Essa condição é definida como uma taxa de crescimento fetal menor que a esperada quando comparado com o tempo de gestação^14^ ^),(^ ^15^ e sua causa mais comum é a insuficiência placentária, que expõe o feto a um ambiente de hipóxia crônica e déficit de aporte nutricional, com risco potencial para o desenvolvimento, em especial, do cérebro^15^ ^),(^ ^16^.

O RCIU tem sido associado a maturação anormal do cérebro^17^ ^),(^ ^18^, a piores resultados cognitivos na infância^12^ e ao QI mais baixo aos 17 anos^19^. Um estudo sueco identificou que a associação entre o RCIU e maior risco de baixo desempenho intelectual é parcialmente mediada por fatores socioeconômicos^20^.

A SES desfavorável parece prejudicar o desenvolvimento cerebral^21^ e intelectual^1^. Existem evidências da relação positiva entre a SES na infância e o QI^22^ e de que ela seja parcialmente mediada por variações anatômicas cerebrais^23^.

Apesar das evidências das alterações cerebrais associadas ao RCIU, sua influência na inteligência, o modo como isso ocorre ao longo do desenvolvimento e a interferência de variáveis ambientais, como a SES, ainda não são eventos completamente compreendidos.

Assim, objetiva-se com este estudo responder às seguintes questões: A SES no nascimento e o RCIU tem efeito direto sobre o QI aos 18 e 19 anos? O efeito da SES no nascimento sobre o QI aos 18 e 19 anos é mediado pelo ganho de peso materno gestacional, RCIU, tempo de aleitamento materno e escolaridade aos 18 e 19 anos? A associação entre o RCIU e o QI aos 18 e 19 anos é mediada pelo baixo peso ao nascer?

## MÉTODOS

### Delineamento do Estudo

Este estudo de coorte realizado com indivíduos nascidos na cidade de São Luís, Maranhão, envolve três períodos diferentes: nascimento, 7 a 9 anos, 18 e 19 anos. Esta coorte faz parte da pesquisa “Determinantes ao longo do ciclo vital da obesidade, precursores de doenças crônicas, capital humano e saúde mental”, desenvolvida pela Universidade Federal do Maranhão (UFMA), Faculdade de Medicina de Ribeirão Preto (Universidade de São Paulo - USP) e Universidade Federal de Pelotas (UFPel).

### População e Amostra do Estudo

A coorte foi iniciada ao nascimento em dez hospitais públicos e privados da cidade, no período de março de 1997 a fevereiro de 1998, incluindo 96,3% dos nascimentos do período por meio de amostragem sistemática com estratificação proporcional de acordo com o número de nascimentos em cada maternidade de um em cada sete partos. Foram excluídos partos múltiplos, natimortos e gemelares. A amostra final totalizou 2.443 nascimentos^24^.

O primeiro seguimento ocorreu quando as crianças estavam com 7 a 9 anos de idade, em 2005 e 2006, por meio de delineamento complexo de amostragem, utilizando a variável peso ao nascer para definir a amostra necessária para a avaliação na idade escolar. A amostra final totalizou 805 crianças nesse seguimento, sendo 673 acompanhadas desde o nascimento e 132 crianças nascidas entre 1997 e 1998 incluídas na coorte retrospectiva^25^.

O segundo seguimento ocorreu entre janeiro e novembro de 2016, quando os sujeitos estavam com 18 e 19 anos de idade. Dos 673 participantes do seguimento anterior que foram acompanhados desde o nascimento, 313 participaram desse seguimento^26^. Foram incluídos no estudo os sujeitos que foram avaliados nas três fases (nascimento, 7 a 9 anos, 18 e 19 anos) e que apresentaram os dados necessários para mensuração do crescimento intrauterino e QI aos 18 a 19 anos.

### Coleta de Dados

Na coleta ao nascimento utilizou-se a Ficha de Registro de Nascimento e foi aplicado um questionário padronizado contendo questões demográficas e antropométricas. A idade gestacional foi calculada a partir da data da última menstruação relatada pela mãe. O peso no início e final da gestação também foi relatado pela mãe.

O peso da criança, sem roupas, foi aferido logo após o nascimento utilizando balança infantil ajustada para 10 gramas. As balanças utilizadas foram verificadas periodicamente e substituídas sempre que foram detectados defeitos. Os recém-nascidos foram medidos entre 12 e 24 horas de vida por meio de um antropômetro do tipo ARTHAG^24^.

O crescimento intrauterino foi classificado com base na razão de peso ao nascimento^27^. Trata-se da razão entre o peso ao nascer e a média de peso para idade gestacional da curva de referência de Williams^28^. Considerou-se RCIU a razão de peso ao nascimento menor que 0,85 e ausência de RCIU a razão de peso ao nascimento maior ou igual a 0,85^27^.

As informações sobre o aleitamento materno foram coletadas durante o primeiro seguimento, aos 7 a 9 anos, por meio de perguntas estruturadas. Para o este estudo considerou-se a duração do aleitamento materno, que faz referência ao tempo em meses no qual a criança foi amamentada sem importar se foi exclusivo ou não.

A inteligência foi avaliada no segundo seguimento, aos 18 e 19 anos, utilizando a Escala de Inteligência de Wechsler para adultos (WAIS-III), que retrata uma medida pontual do nível de inteligência^29^. Nos casos em que o participante apresentava alguma condição que impossibilitasse a realização do teste, ele não foi aplicado.

A WAIS-III é composta por 14 subtestes, divididos em uma Escala Verbal, uma Escala de Execução e uma Escala Total. A Escala Verbal analisa vocabulário, semelhanças, aritmética, dígitos, informação, compreensão e sequência de números-letras. A Escala de Execução reúne códigos, cubos, raciocínio matricial, arranjo de figuras, procura de símbolos, complementação de figuras e organização de objetos. Ela também avalia índices fatoriais, quais sejam: Índice de Compreensão Verbal (vocabulário, semelhanças e informações); Índice de Organização Perceptual (completar figuras, cubos e raciocínio matricial); Índice de Memória Operacional (aritmética, dígitos, sequência de números e letras) e Índice de Velocidade de Processamento (códigos e busca de símbolos) ^(^ ^29^.

A obtenção do QI total ocorreu pela soma dos valores brutos dos subtestes das escalas verbal e de execução, convertidos em resultados ponderados e analisados pela tabela por idade correspondente ao manual WAIS III, refletindo os níveis de inteligência dos participantes. A classificação do QI é dada da seguinte forma: Déficit Intelectual (69 ou menos); Limítrofe (70 a 79); Médio Inferior (80 a 89); Médio (90 a 109); Médio Superior (110 a 119); Superior (120 a 129); Muito Superior (130 ou mais) ^(^ ^29^.

Para caracterização da amostra desteestudo, agrupou-se a classificação do QI em três categorias: abaixo da média da população (≤ 89 pontos); na média da população (90 a 109 pontos); acima da média da população (≥ 110 pontos).

### Variáveis

As variáveis explanatórias observadas no nascimento foram: escolaridade materna e paterna em anos completos (0 a 4; 5 a 8; 9 a 11; 12 ou mais); ocupação do chefe da família (manual não qualificado e desempregado; manual qualificado e semiqualificado; não manual); renda familiar em salários-mínimos (< 1; 1 a 3; > 3) e RCIU: ausente (≥ 0,85) ou presente (< 0,85) ^(^ ^27^.

As demais variáveis do nascimento analisadas foram: idade materna, tratada como numérica contínua na modelagem estatística e como categórica (< 20 anos; 20 a 34 anos; ≥ 35 anos) para caracterização da amostra; ganho de peso gestacional, calculado pela diferença entre o peso no final da gestação e o peso antes da gestação, e tratada como variável numérica contínua na modelagem estatística e como categórica (< 11kg; 11 a 16kg; > 16kg) para caracterização da amostra; baixo peso ao nascer: não (≥ 2500g) ou sim (< 2500g) ^(^ ^25^. O tempo de aleitamento materno foi tratado como variável numérica contínua na modelagem estatística e como categórica (< 2 meses; 2 a 6 meses; > 6 meses) para caracterização da amostra.

A variável escolaridade aos 18 e 19 anos, em anos completos, foi tratada como categórica (0 a 4; 5 a 8; 9 a 11; 12 ou mais). A variável dependente foi o QI aos 18 e 19 anos. Para o modelo teórico testado, a variável foi tratada como numérica contínua e, para caracterização da amostra, como categórica: abaixo da média (≤ 89 pontos); na média (90 a 109 pontos); acima da média (≥ 110 pontos) ^(^ ^29^.

### Análise Estatística

A análise descritiva dos dados foi realizada utilizando a versão 14 do programa Stata (StataCorp.,*CollegeStation*, Estados Unidos da América). As variáveis categóricas foram apresentadas por meio de frequências absolutas e relativas, as variáveis numéricas por média e desvio padrão.

Utilizou-se modelagem de equações estruturais^30^ para investigar a associação do RCIU e SES no nascimento com as variáveis explanatórias e seus efeitos sobre o QI aos 18 e 19 anos. Construiu-se um modelo híbrido, composto por análise fatorial confirmatória, utilizada para construção da variável latente SES no nascimento, e por análise de caminhos, utilizada para analisar os efeitos do RCIU e SES no nascimento no QI aos 18 e 19 anos e estimar as relações lineares entre as variáveis. Para isso utilizou-se a versão 7 do software Mplus.

**Figura 1:**
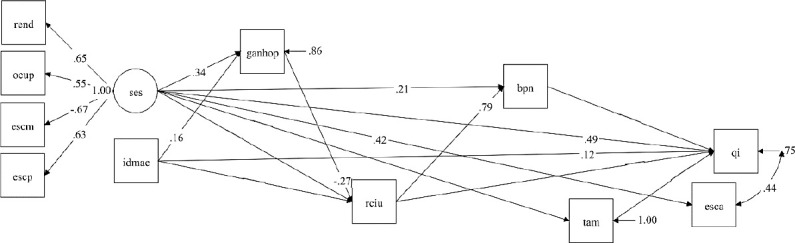
Diagrama de caminhos da associação entre o retardo no crescimento intrauterino, situação socioeconômica no nascimento e o quociente de inteligência aos 18 e 19 anos em participantes da coorte do consórcio RPS de São Luís. São Luís (Maranhão), Brasil, 1997-2016. rend: renda familiar no nascimento; ocup: ocupação do chefe da família no nascimento; escm: escolaridade materna no nascimento; escp: escolaridade paterna no nascimento, ses: situação socioeconômica no nascimento; idmae: idade materna no nascimento; ganhop: ganho de peso gestacional; rciu: retardo no crescimento intrauterino; bpn: baixo peso ao nascer; tam: tempo de aleitamento materno; esca: escolaridade aos 18 e 19 anos; qi: quociente de inteligência aos 18 e 19 anos.

De acordo com o modelo teórico proposto, a variável latente SES no nascimento foi construída a partir da correlação entre as variáveis renda familiar, ocupação do chefe da família, escolaridade materna e paterna observadas no nascimento do participante. Na análise de caminhos, as variáveis SES e idade materna no nascimento do participante ocuparam a posição mais distal e determinaram o ganho de peso materno gestacional, RCIU, baixo peso ao nascer e tempo de aleitamento materno, que determinaram o QI aos 18 e 19 anos. A escolaridade aos 18 e 19 anos ocupou a posição mais proximal no modelo em correlação com o QI (Figura 1).

O estimador utilizado foi o dos mínimos quadrados ponderados robustos ajustados pela média e variância -Weighted Least Squares Mean and Variance Adjusted (WLSMV), empregado para análises de variáveis observadas categóricas. A parametrização THETA foi utilizada para controlar as diferenças de variâncias residuais.

Os dados faltantes foram imputados nas variáveis pelo software Mplus, com base nas variáveis que se apresentaram anteriores a ela no modelo teórico, utilizando análise de frequências e análise Bayesiana^31^. Com a realização da imputação, os dados faltantes não prejudicaram o resultado.

Para avaliar o ajuste do modelo, considerou-se bom o seguinte: a) p-valor superior a 0,05 para o teste qui-quadrado (χ^2^); b) valor do *Root Mean Square Error of Approximation* (RMSEA) inferior a 0,05 e limite superior do intervalo de confiança de 90% inferior a 0,08; c) valores superiores a 0,95 para o Comparative Fit Index (CFI) e o Tucker Lewis Index (TLI); e d) valores Weighted Root Mean Square Residual (WRMR) menores que 1^30^.

Utilizou-se o comando modindices para obtenção de sugestões de alterações das hipóteses iniciais^31^, entretanto, não houve propostas plausíveis do ponto de vista teórico. No modelo final foram avaliados os efeitos totais, diretos e indiretos, das variáveis. Julgou-se haver efeito quando p-valor foi inferior a 0,05.

Para interpretar os efeitos das variáveis no QI, multiplicou-se o valor do coeficiente padronizado do efeito total obtido no modelo estrutural pelo desvio padrão da média do QI.

### Aspectos Éticos

O estudo atendeu aos critérios da Resolução 466/2012^32^ do Conselho Nacional de Saúde do Brasil. Os responsáveis pelos sujeitos assinaram o Termo de Consentimento Livre e Esclarecido (TCLE). Foi facultada a desistência sem qualquer prejuízo para o entrevistado, em qualquer etapa da pesquisa. O projeto e o TCLE da terceira fase da pesquisa foi aprovado pelo Comitê de Ética em Pesquisa do Hospital Universitário da Universidade Federal do Maranhão: Parecer Consubstanciado número 1.302.489 de 29 de outubro de 2015.

## RESULTADOS

Na amostra de 313 jovens, o QI médio foi de 99,52 pontos ± 11,18 pontos, sendo o desempenho mais baixo 70 e o mais alto 137 pontos. A maioria dos participantes (60,38%) obteve pontuação classificada como na média para a população e 77,32% apresentavam 9 a 11 anos de estudo (Tabela 1).

**Tabela 1:** Distribuição do quociente de inteligência aos 18 e 19 anos de acordo com as características socioeconômicas e demográficas no período peri e pós-natal e aos 18 e 19 anos em participantes da coorte do consórcio RPS de São Luís. São Luís (Maranhão), Brasil, 1997-2016.

**Variável *(p-valor)*** ^*a*^	Quociente de inteligência			Total n (%)
	Abaixo da média n (%)	Na média n (%)	Acima da média n (%)	
**Da família no nascimento**				
Idade materna *(0,150)* ^*a*^				
Menos que 20 anos	19 (32,76)	55 (29,10)	10 (15,15)	84 (26,84)
20 a 34 anos	37 (63,79)	128 (67,72)	52 (78,79)	217 (69,33)
35 anos ou mais	02 (3,45)	06 (3,17)	04 (6,06)	12 (3,83)
Escolaridade materna *(<0,001)* ^*a*^				
0 a 4 anos	-	04 (2,12)	03 (4,55)	07 (2,24)
5 a 8 anos	11 (18,97)	79 (41,80)	38 (57,58)	128 (40,89)
9 a 11 anos	31 (53,45)	86 (45,50)	21 (31,82)	138 (44,09)
12 ou mais	16 (27,59)	20 (10,58)	04 (6,06)	40 (12,78)
Escolaridade paterna* *(<0,001)* ^*a*^				
0 a 4 anos	16 (34,04)	20 (12,35)	03 (4,84)	39 (14,39)
5 a 8 anos	19 (40,43)	56 (34,57)	15 (24,19)	90 (33,21)
9 a 11 anos	11 (23,40)	79 (48,77)	41 (66,13)	131 (48,34)
12 ou mais	01 (2,13)	07 (4,32)	03 (4,84)	11 (4,06)
Ocupação do chefe da família* *(0,181)* ^*a*^				
Manual não qualificado e desempregado	23 (41,82)	57 (31,15)	14 (21,54)	94 (31,02)
Manual qualificado e semiqualificado	23 (41,82)	95 (51,91)	36 (55,38)	154 (50,83)
Não manual	09 (16,36)	31 (16,94)	15 (23,08)	55 (18,15)
Renda familiar* *(0,004)* ^*a*^				
Menos que 1 SM	14 (24,56)	24 (13,71)	07 (11,48)	45 (15,36)
1 a menos que 4 SM	22 (38,60)	76 (43,43)	14 (22,95)	112 (38,23)
4 SM ou mais	21 (36,84)	75 (42,86)	40 (65,57)	136 (46,42)
**Do período peri e pós-natal**				
Ganho de peso gestacional *(0,246)* ^*a*^				
Menor que 11kg	29 (50,0)	106 (56,08)	30 (45,45)	165 (52,72)
11 a 16kg	12 (20,69)	47 (24,87)	22 (33,33)	81 (25,88)
Maior que 16kg	17 (29,31)	36 (19,05)	14 (21,21)	67 (21,41)
Retardo no crescimento intrauterino *(0,468)* ^*a*^				
Ausente	50 (86,21)	153 (80,95)	57 (86,36)	260 (83,07)
Presente	08 (13,79)	36 (19,05)	09 (13,64)	53 (16,93)
Baixo peso ao nascer *(0,538)* ^*a*^				
Não	49 (84,48)	167 (88,36)	60 (90,91)	276 (88,18)
Sim	09 (15,52)	22 (11,64)	06 (9,09)	37 (11,82)
Tempo de aleitamento materno* *(0,200)* ^*a*^				
Menor que 02 meses	26 (47,27)	80 (43,48)	29 (44,62)	135 (44,41)
02 a 06 meses	20 (36,36)	87 (47,28)	33 (50,77)	140 (46,05)
Maior que 06 meses	09 (16,36)	17 (9,24)	03 (4,62)	29 (9,54)
**18 e 19 anos**				
Escolaridade *(< 0,001)* ^*a*^				
0 a 4 anos	04 (6,90)	10 (5,29)	-	14 (4,47)
5 a 8 anos	-	04 (2,12)	-	04 (1,28)
9 a 11 anos	53 (91,38)	150 (79,37)	39 (59,09)	242 (77,32)
12 ou mais	01 (1,72)	25 (13,23)	27 (40,91)	53 (16,93)
**Total**	58 (100)	189 (100)	66 (100)	313 (100)

*Excluídos os ignorados; SM: salário-mínimo adotado no Brasil em 1997.

^a^Teste Qui-quadrado.

Durante a gestação, o ganho de peso materno médio foi de 8,84kg ± 5,82kg, 16,93% dos participantes apresentaram RCIU, 11,82% apresentaram baixo peso ao nascer e 46,05% foram amamentados durante 02 a 06 meses (Tabela 1).

A idade média das mães dos participantes, no momento do parto, foi 23,24 anos ± 5,33 anos e 44,09% delas apresentavam 9 a 11 anos de estudo. Entre os pais dos participantes, 48,34% apresentavam 9 a 11 anos de estudo no momento do nascimento. A ocupação dos chefes das famílias era predominantemente manual qualificada ou semiqualificada (50,83%) e 46,42% das famílias apresentavam renda de 4 salários-mínimos ou mais no momento do nascimento dos participantes (Tabela 1).

O modelo proposto para investigar os caminhos da associação entre o RCIU e SES no nascimento e o QI aos 18 e 19 anos apresentou bom ajuste para os indicadores teste qui-quadrado, RMSEA, CFI, TLI e WRMR (Tabela 2) e não houve sugestões plausíveis de modificação.

**Tabela 2:** Índices de ajuste do modelo estrutural da associação entre retardo no crescimento intrauterino e situação socioeconômica no nascimento e o quociente de inteligência aos 18 e 19 anos em participantes da coorte do consórcio RPS de São Luís. São Luís (Maranhão), Brasil, 1997-2016.

Índices de ajuste	Modelo^a^
χ^2b^	50,808
Graus de liberdade	36
p-valor	0,052
RMSEA	0,036
IC 90%	0,000 - 0,058
p-valor	0,838
CFI	0,967
TLI	0,949
WRMR	0,760

RMSEA: Root Mean Square Error of Approximation; CFI: Comparative Fit Index; TLI: Tucker-Lewis Index; WRMR: Weighted Root Mean Square Residual.

^a^ Modelo final com melhor ajuste.

^b^ Teste Qui-quadrado.

A análise fatorial para construção da variável latente SES no nascimento mostrou que as variáveis explanatórias apresentaram correlação com o construto, apresentando valores de p menores que 0,001 e carga fatorial maior que 0,5, exceto a escolaridade materna cuja carga fatorial foi -0,667. As cargas fatoriais e coeficientes padronizados dos efeitos diretos das variáveis explanatórias sobre o QI aos 18 e 19 anos estão listados na Tabela 3 e o diagrama de caminhos do modelo explicativo proposto (Figura 1) expõe somente os coeficientes padronizados das equações cuja associação foi significante (p-valor < 0,05).

**Tabela 3:** Coeficiente padronizado, erro padrão e valores p dos efeitos diretos das variáveis explanatórias no quociente de inteligência aos 18 e 19 anos em participantes da coorte do consórcio RPS de São Luís. São Luís (Maranhão), Brasil, 1997-2016.

Variáveis	Coeficiente padronizado	Erro padrão	p-valor
**Variável latente**			
SES no nascimento			
Renda familiar	0,651	0,064	< 0,001
Ocupação do chefe da família	0,548	0,066	< 0,001
Escolaridade materna	-0,667	0,055	< 0,001
Escolaridade paterna	0,633	0,065	< 0,001
**Efeitos diretos**			
Quociente de inteligência			
SES no nascimento	0,495	0,095	< 0,001
Idade materna	0,116	0,057	0,042
Retardo no crescimento intrauterino	0,382	0,243	0,184
Baixo peso ao nascer	-0,405	0,240	0,092
Tempo de aleitamento materno	-0,107	0,056	0,059
Escolaridade **aos 18 e 19 anos**			
SES no nascimento	0,416	0,091	< 0,001
Tempo de aleitamento materno			
SES no nascimento	0,021	0,065	0,748
Baixo peso ao nascer			
SES no nascimento	0,214	0,098	0,029
Retardo no crescimento intrauterino	0,786	0,064	< 0,001
Retardo no crescimento intrauterino			
Idade materna	-0,134	0,088	0,129
Ganho de peso gestacional	-0,267	0,109	0,014
SES no nascimento	0,027	0,108	0,800
Ganho de peso gestacional			
Idade materna	0,156	0,055	0,004
SES no nascimento	0,344	0,063	< 0,001
**Correlação**			
Quociente de inteligência			
Escolaridade **aos 18 e 19 anos**	0,439	0,084	< 0,001

O RCIU não apresentou efeito total (p-valor = 0,957), direto (p-valor=0,184) ou indireto (p-valor = 0,108) sobre o QI aos 18 e 19 anos e o BPN não mediou a associação entre o RCIU e o QI (p-valor = 0,538) (Tabela 4).

**Tabela 4:** Coeficiente padronizado, erro padrão e valores p dos efeitos total, direto e indireto do retardo no crescimento intrauterino e da situação socioeconômica no nascimento sobre o quociente de inteligência aos 18 e 19 anos em participantes da coorte do consórcio RPS de São Luís. São Luís (Maranhão), Brasil, 1997-2016.

Caminhos	Coeficiente padronizado	Erro padrão	p-valor
**Efeitos totais, diretos e indiretos**			
SES no nascimento			
efeito total	0,406	0,059	< 0,001
efeito direto	0,495	0,095	< 0,001
efeito indireto	-0,089	0,077	0,248
Retardo no crescimento intrauterino			
efeito total	0,004	0,080	0,957
efeito direto	0,323	0,243	0,184
efeito indireto	-0,318	0,198	0,108

A SES no nascimento apresentou efeito total positivo de 0,406 desvio-padrão (p-valor < 0,001) sobre a média do QI, que corresponde a uma elevação de 4,54 pontos a cada elevação do nível socioeconômico no nascimento (Tabela 4). Esse efeito não foi mediado pelas demais variáveis explanatórias inclusas no modelo (Figura 1).

Identificou-se também correlação positiva (CP = 0,439; p-valor < 0,001) entre a escolaridade do participante e seu QI aos 18 e 19 anos, e efeito direto positivo de 0,416 (p-valor < 0,001) da SES no nascimento sobre a escolaridade do participante aos 18 e 19 anos (Tabela 3). Entretanto, a escolaridade do participante não mediou a relação entre a SES no nascimento e o QI aos 18 e 19 anos (Tabela 4).

Adicionalmente, a idade materna ao nascimento do participante apresentou efeito positivo direto de 0,116 desvio-padrão (p-valor = 0,042) sobre a média do QI aos 18 e 19 anos (Tabela 3), correspondendo ao incremento de 1,30 pontos no QI a cada elevação de 1 ano na idade materna no nascimento.

## DISCUSSÃO

Neste estudo, o RCIU não apresentou efeito direto ou indireto sobre o QI aos 18 e 19 anos e o BPN não foi mediador dessa associação. A SES no nascimento gerou efeito positivo de longo prazo na inteligência, elevando a pontuação do QI dos participantes. Esse efeito não foi mediado pelo ganho de peso materno gestacional, RCIU e tempo de aleitamento materno.

A associação entre o RCIU e o desenvolvimento intelectual ainda não está completamente explicada pela ciência. Apesar das evidências de que o RCIU está associado a prejuízos na maturação cerebral^33^ ^),(^ ^34^ e na cognição na infância^35^, na amostra estudada esse efeito não foi observado.

Assim como nesteestudo, o RCIU não influenciou o QI entre participantes de uma coorte dinamarquesa^36^, sugerindo que essa condição intrauterina adversa pode não impactar no desenvolvimento intelectual até a idade adulta.

É possível que o resultado deste estudo seja parcialmente explicado pela fisiopatologia que envolve o RCIU. A insuficiência na circulação placentária, principal causa dessa restrição do crescimento, expõe o feto a uma condição de hipoxemia crônica ou privação de nutrientes que prejudicam seu desenvolvimento. Existem evidências de que, diante dessa adversidade, o feto redistribui seu débito cardíaco para priorizar o suprimento de oxigênio e nutrientes para o cérebro^16^. Assim, é possível que a adaptação da circulação fetal tenha preservado o desenvolvimento cerebral dos participantes deste estudo.

Em contrapartida, a SES no nascimento dos participantes deste estudo apresentou efeito positivo no seu QI aos 18 e 19 anos. A influência da SES da família no desenvolvimento intelectual ao longo da vida tem sido evidenciada em diversos estudos^1^ ^),(^ ^22^ ^),(^ ^37^ ^),(^ ^38^, sendo associada a maiores níveis de inteligência em várias etapas do desenvolvimento^3^ ^),(^ ^6^ ^),(^ ^7^.

Fatores como renda familiar, educação parental e qualidade do ambiente no início da vida desempenham papéis críticos no desenvolvimento cognitivo^39^ e, provavelmente, o conjunto desses fatores contribuiu para os resultados do presente estudo.

Uma das hipóteses que explicaria esse resultado está na influência da qualidade do ambiente em que a criança vive. Famílias em SES desfavorável frequentemente enfrentam níveis mais altos de estresse, incluindo insegurança alimentar, moradia precária e incertezas financeiras^40^, condições de estresse crônico podem prejudicar o desenvolvimento do cérebro durante a infância^41^.

Além disso, a qualidade dos estímulos ofertados à criança parece ser determinante para seu desenvolvimento intelectual e, nessa perspectiva, a escolaridade dos pais se destaca entre os indicadores da SES^37^.

Para alguns pesquisadores, o nível educacional dos pais é determinado, de forma mais expressiva, por sua inteligência individual. Nessa perspectiva, a escolaridade dos pais estaria associada à inteligência dos filhos devido ao compartilhamento de genes envolvidos no desenvolvimento da capacidade intelectual^42^.

É importante considerar também o componente ambiental da relação entre escolaridade parental e inteligência dos filhos. Pais com maior escolaridade parecem ter maiores chances de gerar rendas mais altas^43^ e oferecer ambientes intelectualmente mais estimulantes que, possivelmente, resultariam em desempenho superior em alguns testes^44^. Além disso, eles parecem destinar mais atenção e tempo à educação dos filhos, e investir mais dinheiro em serviços direcionados a melhoria do seu desempenho escolar^45^ ^),(^ ^46^. Consequentemente, famílias com maior nível de educação parental costumam apresentar SES mais elevada e promover maior acesso à educação para seus filhos, o que poderia explicar o efeito positivo da SES no nascimento dos participantes deste estudo na sua escolaridade aos 18 e 19 anos.

A inteligência e escolaridade apresentaram correlação positiva na amostra estudada, inexistindo caminhos causais unidirecionais entre elas. Essa relação pode ser explicada pela influência dos componentes genético e ambiental envolvidos no desenvolvimento das duas variáveis, já discutido anteriormente.

A relação observada entre a idade materna e o QI do filho, revelou que o QI dos participantes cujas mães apresentavam idades mais avançadas no seu nascimento foi maior que o daqueles cujas mães eram mais jovens.

É possível que essa associação se relacione com o ambiente e a SES, tendo em vista que mulheres com mais idade frequentemente possuem maior estabilidade financeira e níveis mais elevados de educação, o que pode ter favorecido o desenvolvimento intelectual dos seus filhos. Além disso, mães mais velhas são capazes de fornecer um ambiente mais enriquecido em termos de estímulos cognitivos e emocionais, conforme demonstrado em um estudo realizado na China^47^, o que também pode ter contribuído para o melhor desempenho intelectual dos participantes deste estudo.

Uma limitação deste estudo foi a perda de seguimento dos sujeitos, devido ao longo período temporal entre os seguimentos e à dificuldade de localização dos jovens, apesar de todas as estratégias de busca utilizadas. Com uma amostra maior, presume-se a possibilidade de estimar outros efeitos de determinantes importantes.

Um ponto forte é o tipo do estudo. Estudos de coorte tem vantagens em relação à causalidade reversa e a possibilidade de acompanhamento da mesma população. Além disso, o método estatístico utilizado, modelagem de equações estruturais, confere robustez a análise e oportuniza lidar simultaneamente com várias relações de dependência, podendo representar variáveis latentes nessas relações, além de modelar o erro de medição no processo de estimativa^30^.

O QI não foi influenciado pelo RCIU na amostra estudada, mas sofreu efeito direto da SES no nascimento, que, quando em níveis mais elevados, associou-se à maior pontuação aos 18 e 19 anos. Esses resultados sugerem que os fatores ambientais, como a SES, aos quais as crianças são expostas podem ser determinantes importantes no seu desenvolvimento intelectual. É possível afirmar, no entanto, a necessidade de mais estudos que auxiliem a compreensão dos caminhos complexos que envolvem o desenvolvimento da inteligência.
