# Supplementary material for: Adversidades no período intrauterino e neonatal interferem na inteligência?
Source: Rev Saude Publica. 2025 Jun 16;59:e15. [Article in Portuguese] doi: 10.11606/s1518-8787.2025059006206 (PMC12169698; doi:10.11606/s1518-8787.2025059006206)
Supplement: Translation

# Translation: Do adversities in the intrauterine and neonatal periods interfere with intelligence?

**DOI:** 10.11606/s1518-8787.2025059006206

**Authors:** Janielle Ferreira de Brito Lima, Rosângela Fernandes Lucena Batista, Liliana Yanet Gómez Aristizábal, Cecilia Claudia Costa Ribeiro de Almeida, Vanda Maria Ferreira Simões, Antônio Augusto Moura da Silva

**Affiliations:** I Universidade Federal do Maranhão. Departamento de Enfermagem. São Luís, MA, Brasil.; II Universidade Federal do Maranhão. Departamento de Saúde Pública. São Luís, MA, Brasil.

**Keywords:** Fetal Growth Retardation, Socioeconomic Factors, Intelligence

## Abstract

**OBJECTIVE:**

Investigate the effects of intrauterine growth restriction and socioeconomic status at birth on intellectual development.

**METHODS:**

Study conducted with 313 participants from a birth cohort from São Luís, in the state of Maranhão, assessed at birth and between 18 and 19 years of age. Variables at birth and early years of life (maternal age, maternal education, paternal education, occupation of the head of the household, family income, maternal gestational weight gain, intrauterine growth restriction, low birth weight, and breastfeeding duration), and education at 18 and 19 years were tested as determinants of intelligence quotient at 18 and 19 years using structural equation modeling.

**RESULTS:**

Results: Intrauterine growth restriction did not have a total (p-value = 0.957), direct (p-value = 0.184), or indirect (p-value = 0.108) effect on the intelligence quotient at 18 and 19 years of age. Socioeconomic status at birth had a total positive effect of 0.406 standard deviations (p-value < 0.001) on its mean, corresponding to an increase of 4.54 points in the quotient for each increase in socioeconomic level at birth. This effect was not mediated by the other explanatory variables included in the model; however, a direct positive effect of 0.416 (p-value < 0.001) of socioeconomic status on education was identified, which showed a positive correlation (PC = 0.439; p-value < 0.001) with intelligence. Maternal age at birth also had a direct positive effect of 0.116 standard deviations (p-value = 0.042) on the mean intelligence quotient (QI) at 18 and 19 years, corresponding to an increase of 1.30 points for each 1-year increase in maternal age at birth.

**CONCLUSION:**

Higher socioeconomic levels at birth directly affected the participant’s intelligence and education positively, increasing the mean QI and education level at 18 and 19 years. A positive correlation was also identified between the two variables.

## INTRODUCTION

Intellectual development has been widely studied due to its impact on socially relevant outcomes, such as professional and personal successes^1^ ^)-(^ ^3^. Intelligence development appears to involve genetic^4^, neurophysiological^5^, environmental^6^ ^),(^ ^7^, and adverse experiences from conception to adulthood^8^ ^)-(^ ^10^.

Adversities experienced during the intrauterine and neonatal periods have been associated with intellectual development throughout life. Variables such as family socioeconomic status (SES)^6^, breastfeeding^11^, low birth weight^12^, and intrauterine growth restriction (IUGR) ^(^ ^12^ ^),(^ ^13^ appear to interfere with neurological development and intelligence levels.

Among these adversities, IUGR stands out for its risks to brain formation and maturation^13^. This condition is defined as a fetal growth rate lower than expected compared to the gestational age^14^ ^),(^ ^15^. Its most common cause is placental insufficiency, which exposes the fetus to an environment of chronic hypoxia and nutritional deficiency, with potential risks to development, especially of the brain^15^ ^),(^ ^16^.

IUGR has been associated with abnormal brain maturation^17^ ^),(^ ^18^, worse cognitive outcomes in childhood^12^, and lower IQ at age 17^19^. A Swedish study found that the association between IUGR and a higher risk of low intellectual performance is partially mediated by socioeconomic factors^20^.

Unfavorable SES appears to impair brain^21^ and intellectual^1^ development. There is evidence of a positive relationship between SES in childhood and IQ^22^ and that anatomical variations in the brain partially mediate it^23^.

Despite the evidence of brain changes associated with IUGR, its influence on intelligence, how this occurs throughout development, and the interference of environmental variables, such as SES, are still not fully understood.

Thus, this study aims to answer the following questions: Do SES at birth and IUGR directly affect IQ at 18 and 19? Is the effect of SES at birth on IQ at 18 and 19 years of age mediated by maternal gestational weight gain, IUGR, breastfeeding duration, and education at 18 and 19? Does low birth weight mediate the association between IUGR and QI at 18 and 19?

## METHODS

### Study Design

This cohort study was conducted with individuals born in the city of São Luís, in the state of Maranhão, and involved three different periods: birth, 7 to 9 years, and 18 and 19 years. This cohort is part of the research “*Determinantes ao longo do ciclo vital da obesidade, precursores de doenças crônicas, capital humano e saúde mental*“ [Life-cycle determinants of obesity, precursors of chronic diseases, human capital, and mental health], developed by the Universidade Federal do Maranhão (UFMA), Ribeirão Preto Medical School (University of São Paulo - USP), and Federal University of Pelotas (UFPel).

### Study Population and Sample

The cohort was initiated at birth in ten public and private hospitals in the city from March 1997 to February 1998. It included 96.3% of births through systematic sampling with proportional stratification according to the number of births in each maternity hospital, one in seven births. Multiple births, stillbirths, and twins were excluded. The final sample totaled 2,443 births^24^.

The first follow-up occurred when the children were 7 to 9 years old, in 2005 and 2006, through a complex sampling design, using the birth weight variable to define the sample required for the evaluation at school age. The final sample totaled 805 children in this follow-up, of which 673 were followed since birth, and 132 children born between 1997 and 1998 were included in the retrospective cohort^25^.

The second follow-up occurred between January and November 2016, when the subjects were 18 and 19. Of the 673 participants in the previous follow-up who were monitored since birth, 313 participated in this follow-up^26^. The study included subjects evaluated in the three phases (birth, 7 to 9 years, 18 and 19 years) and who presented the necessary data for measuring intrauterine growth and IQ at 18 to 19.

### Data Collection

The data collection used the Birth Registration Form at birth, applying a standardized questionnaire containing demographic and anthropometric questions. Gestational age was calculated from the date of the last menstrual period reported by the mother. The mother also reported weight at the beginning and end of pregnancy.

The child’s weight, without clothes, was measured immediately after birth using a baby scale adjusted to 10 grams. The scales used were checked periodically and replaced whenever defects were detected. Newborns were measured between 12 and 24 hours of life using an ARTHAG anthropometer^24^.

Intrauterine growth was classified based on the birth weight ratio^27^. This is the ratio between birth weight and the average weight for gestational age of the Williams reference curve^28^. IUGR was considered a birth weight ratio of less than 0.85, and the absence of IUGR was a birth weight ratio greater than or equal to 0.85^27^.

Information on breastfeeding was collected through structured questions during the first follow-up at 7 to 9 years of age. This study considered the breastfeeding duration, which refers to the time in months during which the child was breastfed, regardless of whether it was exclusive.

Intelligence was assessed in the second follow-up, at 18 and 19 years of age, using the Wechsler Adult Intelligence Scale (WAIS-III), which provides a specific measure of intelligence level^29^. The test was not administered to participants with conditions that prevented them from taking it.

The WAIS-III comprises 14 subtests, divided into a Verbal Scale, a Performance Scale, and a Full Scale. The Verbal Scale analyzes vocabulary, similarities, arithmetic, digit span, information, comprehension, and letter-number sequencing. The Performance Scale includes digit symbol-coding, block design, matrix reasoning, picture completion, symbol search, picture arrangement, and object assembly. It also evaluates factorial indices, namely, the Verbal Comprehension Index (vocabulary, similarities, and information); Perceptual Organization Index (picture completion, block design, and matrix reasoning); Working Memory Index (arithmetic, digit span, and letter-number sequencing), and Processing Speed Index (digit symbol-coding and symbol search) ^(^ ^29^.

The total IQ was obtained by adding the raw values of the subtests of the verbal and performance scales, converting them into weighted results, and analyzing using the age table corresponding to the WAIS-III manual, reflecting the intelligence levels of the participants. The IQ classification is given as follows: Intellectual Deficit (69 or less); Borderline (70 to 79); Low Average (80 to 89); Average (90 to 109); High Average (110 to 119); Superior (120 to 129); Very Superior (130 or above) ^(^ ^29^.

The IQ classification was grouped into three categories to characterize the sample of this study: below the population average (≤ 89 points), at the population average (90 to 109 points), and above the population average (≥ 110 points).

### Variables

The explanatory variables observed at birth were maternal and paternal education in complete years (0 to 4; 5 to 8; 9 to 11; 12 or more); occupation of the head of the household (unskilled manual and unemployed; skilled and semi-skilled manual; non-manual); family income in minimum wages (SM) (< 1; 1 to 3; > 3), and IUGR: absent (≥ 0.85) or present (< 0.85) ^(^ ^27^.

The other birth variables analyzed were maternal age, treated as a continuous numeric in the statistical modeling and as categorical (< 20 years; 20 to 34 years; ≥ 35 years) to characterize the sample; gestational weight gain, calculated by the difference between the weight at the end of pregnancy and the weight before pregnancy, and treated as a continuous numerical variable in the statistical modeling and as categorical (< 11 kg; 11 to 16 kg; > 16 kg) for sample characterization; low birth weight: no (≥ 2500 g) or yes (< 2500 g) ^(^ ^25^. Breastfeeding duration was treated as a continuous numerical variable in the statistical modeling and as categorical (< 2 months; 2 to 6 months; > 6 months) for sample characterization.

The variable of education at 18 and 19 years of age, in complete years, was treated as categorical (0 to 4; 5 to 8; 9 to 11; 12 or more). The dependent variable was QI at 18 and 19 years of age. For the theoretical model tested, the variable was treated as a continuous numeric variable and, for characterization of the sample, as categorical: below average (≤ 89 points); average (90 to 109 points); above average (≥ 110 points) ^(^ ^29^.

### Statistical Analysis

The descriptive analysis of the data was performed using version 14 of the Stata program (StataCorp., College Station, United States of America). The categorical variables were presented using absolute and relative frequencies, and the numerical variables were presented using means and standard deviations.

Structural equation modeling^30^ was used to investigate the association of IUGR and SES at birth with the explanatory variables and their effects on IQ at 18 and 19. A hybrid model was constructed, consisting of confirmatory factor analysis, used to build the latent variable SES at birth, and path analysis, used to analyze the effects of IUGR and SES at birth on IQ at 18 and 19 years of age and to estimate the linear relationships between the variables. Version 7 of the Mplus software was used for this purpose.

According to the proposed theoretical model, the latent variable SES at birth was constructed based on the correlation between the variables of family income, occupation of the head of the household, and maternal and paternal education observed at the participant’s birth. In the path analysis, the variables SES and maternal age at the participant’s birth occupied the most distal position and determined maternal gestational weight gain, IUGR, low birth weight, and breastfeeding duration, which determined IQ at 18 and 19 years. Education at 18 and 19 years occupied the most proximal position in the model in correlation with IQ (Figure 1).

**Figure 1:** Path diagram of the association between intrauterine growth retardation, socioeconomic status at birth, and intelligence quotient at 18 and 19 years in participants of the RPS consortium cohort of São Luís. São Luís (Maranhão), Brazil, 1997-2016. FIB: family income at birth; OCUP: occupation of the head of the household at birth; MEB: maternal education at birth; PEB: paternal education at birth, SES: socioeconomic status at birth; MAB: maternal age at birth; GWG: gestational weight gain; IUGR: intrauterine growth restriction; LBW: low birth weight; BFD: breastfeeding duration; EDUC: education at 18 and 19 years; IQ: intelligence quotient at 18 and 19 years.

The estimator used was the robust weighted least squares adjusted by mean and variance - Weighted Least Squares Mean and Variance Adjusted (WLSMV), used to analyze categorical observed variables. THETA parameterization was used to control differences in residual variances.

The Mplus software imputed missing data to the variables based on the variables that appeared before it in the theoretical model, using frequency and Bayesian analyses^31^. With the imputation performed, the missing data did not affect the result.

When assessing the model’s fit, the following were considered good: a) p-value greater than 0.05 for the chi-square test (χ^2^); b) Root Mean Square Error of Approximation (RMSEA) value less than 0.05 and upper limit of the 90% confidence interval less than 0.08; c) values greater than 0.95 for the Comparative Fit Index (CFI) and the Tucker Lewis Index (TLI); and d) Weighted Root Mean Square Residual (WRMR) values less than 1^30^.

The MODINDICES command was used to obtain suggestions for changes to the initial hypotheses^31^. However, no plausible proposals were presented from a theoretical point of view. The final model assessed the variables’ total, direct, and indirect effects. An effect was considered to exist when the p-value was less than 0.05.

To interpret the variables’ effects on IQ, the value of the standardized coefficient of the total effect obtained in the structural model was multiplied by the standard deviation of the mean IQ.

### Ethical Aspects

The study met the criteria of Resolution 466/2012^32^ of the Brazilian National Health Council. The subjects’ guardians signed the informed consent form. The interviewee was allowed to withdraw without harming the interviewee at any research stage. The project and the consent form of the third phase of the research were approved by the Ethics Committee of the University Hospital of the Federal University of Maranhão: Consolidated Opinion number 1,302,489 of October 29, 2015.

## RESULTS

In the sample of 313 young people, the average IQ was 99.52 points ± 11.18 points, with the lowest performance being 70 and the highest 137 points. Most participants (60.38%) scored average for the population, and 77.32% had 9 to 11 years of education (Table 1).

**Table 1:** Distribution of intelligence quotient at 18 and 19 years according to socioeconomic and demographic characteristics in the peri- and postnatal period and at 18 and 19 years in participants of the RPS consortium cohort of São Luís. São Luís (Maranhão), Brazil, 1997-2016.

**Characteristic *(p-value)*** ^*a*^	Intelligence quotient			Total n (%)
	Below average n (%)	Average n (%)	Above average n (%)	
**Family at birth**				
Maternal age *(0.150)* ^*a*^				
Less than 20 years	19 (32.76)	55 (29.10)	10 (15.15)	84 (26.84)
20 to 34 years	37 (63.79)	128 (67.72)	52 (78.79)	217 (69.33)
35 years or older	02 (3.45)	06 (3.17)	04 (6.06)	12 (3.83)
Maternal education *(<0.001)* ^*a*^				
0 to 4 years	-	04 (2.12)	03 (4.55)	07 (2.24)
5 to 8 years	11 (18.97)	79 (41.80)	38 (57.58)	128 (40.89)
9 to 11 years	31 (53.45)	86 (45.50)	21 (31.82)	138 (44.09)
12 or older	16 (27.59)	20 (10.58)	04 (6.06)	40 (12.78)
Paternal education* *(<0.001)* ^*a*^				
0 to 4 years	16 (34.04)	20 (12.35)	03 (4.84)	39 (14.39)
5 to 8 years	19 (40.43)	56 (34.57)	15 (24.19)	90 (33.21)
9 to 11 years	11 (23.40)	79 (48.77)	41 (66.13)	131 (48.34)
12 or older	01 (2.13)	07 (4.32)	03 (4.84)	11 (4.06)
Occupation of the head of the household* (0.181) ^a^				
Unskilled manual and unemployed	23 (41.82)	57 (31.15)	14 (21.54)	94 (31.02)
Skilled and semi-skilled manual	23 (41.82)	95 (51.91)	36 (55.38)	154 (50.83)
Non-manual	09 (16.36)	31 (16.94)	15 (23.08)	55 (18.15)
Family income* *(0.004)* ^*a*^				
Less than 1 MW	14 (24.56)	24 (13.71)	07 (11.48)	45 (15.36)
1 to less than 4 MW	22 (38.60)	76 (43.43)	14 (22.95)	112 (38.23)
4 MW or more	21 (36.84)	75 (42.86)	40 (65.57)	136 (46.42)
**Peri and postnatal period**				
Gestational weight gain *(0.246)* ^*a*^				
Less than 11 kg	29 (50.0)	106 (56.08)	30 (45.45)	165 (52.72)
11 to 16 kg	12 (20.69)	47 (24.87)	22 (33.33)	81 (25.88)
More than 16 kg	17 (29.31)	36 (19.05)	14 (21.21)	67 (21.41)
Intrauterine growth restriction *(0.468)* ^*a*^				
Absent	50 (86.21)	153 (80.95)	57 (86.36)	260 (83.07)
Present	08 (13.79)	36 (19.05)	09 (13.64)	53 (16.93)
Low birth weight *(0.538)* ^*a*^				
No	49 (84.48)	167 (88.36)	60 (90.91)	276 (88.18)
Yes	09 (15.52)	22 (11.64)	06 (9.09)	37 (11.82)
Breastfeeding duration* *(0.200)* ^*a*^				
Less than 02 months	26 (47.27)	80 (43.48)	29 (44.62)	135 (44.41)
02 to 06 months	20 (36.36)	87 (47.28)	33 (50.77)	140 (46.05)
More than 06 months	09 (16.36)	17 (9.24)	03 (4.62)	29 (9.54)
**18 and 19 years old**				
Education *(<0.001)* ^*a*^				
0 to 4 years	04 (6.90)	10 (5.29)	-	14 (4.47)
5 to 8 years	-	04 (2.12)	-	04 (1.28)
9 to 11 years	53 (91.38)	150 (79.37)	39 (59.09)	242 (77.32)
12 or more	01 (1.72)	25 (13.23)	27 (40.91)	53 (16.93)
**Total**	58 (100)	189 (100)	66 (100)	313 (100)

* Excluded the unknown; MW: minimum wage adopted in Brazil in 1997.

^a^ Chi-square test.

During pregnancy, the average maternal weight gain was 8.84kg ±5.82kg, 16.93% of the participants had IUGR, 11.82% had low birth weight, and 46.05% were breastfed for 02 to 06 months (Table 1).

The average age of the participants’ mothers at the time of delivery was 23.24 years ± 5.33 years, and 44.09% had 9 to 11 years of education. Among the participants’ fathers, 48.34% had 9 to 11 years of schooling at birth. The occupation of the heads of households was predominantly skilled or semi-skilled manual labor (50.83%), and 46.42% of the families had an income of 4 minimum wages or more at the time of the participant’s birth (Table 1).

The model proposed to investigate the paths of association between IUGR and SES at birth and IQ at 18 and 19 years showed a good fit for the chi-square test, RMSEA, CFI, TLI, and WRMR (Table 2), with no plausible suggestions for modification.

**Table 2:** Fit indices of the structural model of the association between intrauterine growth restriction and socioeconomic status at birth and the intelligence quotient at 18 and 19 years in participants of the RPS consortium cohort of São Luís. São Luís (Maranhão), Brazil, 1997-2016.

Fit indices	Model^a^
χ^2b^	50.808
Degrees of freedom	36
p-value	0.052
RMSEA	0.036
90%CI	0.000 - 0.058
p-value	0.838
CFI	0.967
TLI	0.949
WRMR	0.760

RMSEA: Root Mean Square Error of Approximation; CFI: Comparative Fit Index; TLI: Tucker-Lewis Index; WRMR: Weighted Root Mean Square Residual.

^a^ Modelo final com melhor ajuste.

^b^ Chi-square test.

The factor analysis for constructing the latent variable SES at birth showed that the explanatory variables were correlated with the construct, with p-values lower than 0.001 and factor loading higher than 0.5, except for maternal education, whose factor loading was -0.667. The factor loadings and standardized coefficients of the direct effects of the explanatory variables on IQ at 18 and 19 years are listed in Table 3, and the path diagram of the proposed explanatory model (Figure 1) shows only the standardized coefficients of the equations whose association was significant (p-value < 0.05).

**Table 3:** Standardized coefficient, standard error, and p-values of the direct effects of the explanatory variables on intelligence quotient at 18 and 19 years in participants of the RPS consortium cohort of São Luís. São Luís (Maranhão), Brazil, 1997-2016.

Variables	Standardized coefficient	Standard error	p-value
**Latent variable**			
SES at birth			
Family income	0.651	0.064	< 0.001
Occupation of the head of the household	0.548	0.066	< 0.001
Maternal education	-0.667	0.055	< 0.001
Paternal education	0.633	0.065	< 0.001
**Direct effects**			
Intelligence quotient			
SES at birth	0.495	0.095	< 0.001
Maternal age	0.116	0.057	0.042
Intrauterine growth retardation	0.382	0.243	0.184
Low birth weight	-0.405	0.240	0.092
Breastfeeding duration	-0.107	0.056	0.059
Education **at 18 and 19 years**			
SES at birth	0.416	0.091	< 0.001
Breastfeeding duration			
SES at birth	0.021	0.065	0.748
Low birth weight			
SES at birth	0.214	0.098	0.029
Intrauterine growth retardation	0.786	0.064	< 0.001
Intrauterine growth retardation			
Maternal age	-0.134	0.088	0.129
Gestational weight gain	-0.267	0.109	0.014
SES at birth	0.027	0.108	0.800
Gestational weight gain			
Maternal age	0.156	0.055	0.004
SES at birth	0.344	0.063	< 0.001
**Correlation**			
Intelligence quotient			
Education **at 18 and 19 years**	0.439	0.084	< 0.001

**SES:** socioeconomic status

IUGR did not present a total (p-value = 0.957), direct (p-value = 0.184), or indirect effect (p-value = 0.108) on IQ at 18 and 19 years, and LBW did not mediate the association between IUGR and IQ (p-value = 0.538) (Table 4).

**Table 4:** Standardized coefficient, standard error, and p-values of the total, direct, and indirect effects of intrauterine growth retardation and socioeconomic status at birth on intelligence quotient at 18 and 19 years in participants of the RPS consortium cohort of São Luís. São Luís (Maranhão), Brazil, 1997-2016.

Paths	Standardized coefficient	Standard error	p-value
**Total, direct, and indirect effects**			
SES at birth			
total effect	0.406	0.059	< 0.001
direct effect	0.495	0.095	< 0.001
indirect effect	-0.089	0.077	0.248
Intrauterine growth retardation			
total effect	0.004	0.080	0.957
direct effect	0.323	0.243	0.184
indirect effect	-0.318	0.198	0.108

SES at birth presented a positive total effect of 0.406 standard deviations (p-value < 0.001) on the mean IQ, which corresponds to an increase of 4.54 points for each increase in socioeconomic level at birth (Table 4). which corresponds to a rise of 4.54 points for each increase in socioeconomic level (Figure 1).

A positive correlation (PC = 0.439; p-value < 0.001) was also identified between the participant’s education level and their QI at 18 and 19 years of age, and a direct positive effect of 0.416 (p-value < 0.001) of SES at birth on the participant’s education level at 18 and 19 years of age (Table 3). However, the participant’s education level did not mediate the relationship between SES at birth and QI at 18 and 19 years of age (Table 4).

In addition, the participant’s maternal age at birth had a direct positive effect of 0.116 standard deviations (p-value = 0.042) on the mean QI at 18 and 19 years of age (Table 3), corresponding to an increase of 1.30 points in QI for each 1-year increase in maternal age at birth.

## DISCUSSION

In this study, IUGR did not directly or indirectly affect IQ at 18 and 19 years of age, and LBW was not a mediator of this association. SES at birth generated a positive long-term effect on intelligence, increasing the participants’ QI scores. This effect was not mediated by maternal gestational weight gain, IUGR, and breastfeeding duration.

Science has not yet thoroughly explained the association between IUGR and intellectual development. Despite evidence that IUGR is associated with impairments in brain maturation^33^ ^),(^ ^34^ and cognition in childhood^35^, this effect was not observed in the sample studied.

As in this study, IUGR did not influence QI among participants in a Danish cohort^36^, suggesting that this adverse intrauterine condition may not impact intellectual development until adulthood.

The result of this study may be partially explained by the pathophysiology involving IUGR. Insufficient placental circulation, the leading cause of this growth restriction, exposes the fetus to chronic hypoxemia or nutrient deprivation that impairs the development. There is evidence that, when faced with this adversity, the fetus redistributes its cardiac output to prioritize the supply of oxygen and nutrients to the brain^16^. Thus, it is possible that fetal circulation adaptation preserved the brain development of the participants in this study.

In contrast, the SES at birth of the participants in this study positively affected their IQ at 18 and 19 years of age. The influence of family SES on intellectual development throughout life has been demonstrated in several studies^1^ ^),(^ ^22^ ^),(^ ^37^ ^),(^ ^38^ and is associated with higher levels of intelligence at various stages of development^3^ ^),(^ ^6^ ^),(^ ^7^.

Factors such as family income, parental education, and environmental quality early in life play critical roles in cognitive development^39^. All of these factors probably contributed to the present study’s results.

One hypothesis that would explain this result is that the quality of the child’s environment influences it. Families with low SES often face higher levels of stress, including food insecurity, precarious housing, and financial uncertainty^40^. Chronic stress conditions can impair brain development during childhood^41^.

Furthermore, the quality of stimuli offered to children appears to be a determining factor in their intellectual development, and from this perspective, parents’ education stands out among SES indicators^37^.

Some researchers believe that individual intelligence more significantly determines parents’ educational level. From this perspective, parents’ education is associated with their children’s intelligence due to the sharing of genes involved in developing intellectual capacity^42^.

It is also essential to consider the environmental component of the relationship between parental education and children’s intelligence. Parents with higher education levels appear to be more likely to generate higher incomes^43^ and provide more intellectually stimulating environments, which could result in higher performance on some tests^44^. In addition, they appear to devote more attention and time to their children’s education and invest more money in services to improve their children’s academic performance^45^ ^),(^ ^46^. Consequently, families with higher parental education levels tend to have higher SES and promote greater access to education for their children. This could explain the positive effect of SES at birth on the education of the participants in this study at ages 18 and 19.

Intelligence and education showed a positive correlation in the sample studied, with no unidirectional causal paths between them. As previously discussed, this relationship can be explained by the influence of the genetic and environmental components involved in the development of the two variables.

The relationship between maternal age and the child’s IQ revealed that the IQ of participants whose mothers were older at birth was higher than that of younger mothers.

This association may be related to the environment and SES, given that older women often have greater financial stability and higher levels of education, which may have favored their children’s intellectual development. Furthermore, older mothers can provide a more enriched environment in terms of cognitive and emotional stimuli, as demonstrated in a study conducted in China^47^, which may also have contributed to the better intellectual performance of the participants in this study.

A limitation of this study was the loss of follow-up of subjects due to the long period between follow-ups and the difficulty in locating young people despite all the search strategies used. With a larger sample, it is assumed that estimating other effects of essential determinants is possible.

A vital point is the type of study. Cohort studies have advantages concerning reverse causality and the possibility of monitoring the same population. In addition, the statistical method used, structural equation modeling, provides robustness to the analysis and makes it possible to simultaneously deal with several dependency relationships, which can represent latent variables in these relationships, in addition to modeling the measurement error in the estimation process^30^.

IUGR did not influence IQ in the sample studied but was directly affected by SES at birth, which, when at higher levels, was associated with higher scores at 18 and 19 years of age. These results suggest that environmental factors, such as SES, to which children are exposed may be essential determinants in their intellectual development. However, it is possible to affirm the need for more studies that help understand the complex pathways involved in intelligence development.
